# Prognostic Factors for Advanced Pancreatic Cancer Treated with Gemcitabine Plus S-1: Retrospective Analysis and Development of a Prognostic Model

**DOI:** 10.3390/cancers11010057

**Published:** 2019-01-09

**Authors:** Ching-Fu Chang, Pei-Wei Huang, Jen-Shi Chen, Yen-Yang Chen, Chang-Hsien Lu, Pei-Hung Chang, Yu-Shin Hung, Wen-Chi Chou

**Affiliations:** 1 Division of Hematology-Oncology, Department of Internal Medicine, Chang Gung Memorial Hospital at Linkou and Chang Gung University College of Medicine, Taoyuan 333, Taiwan; mr0826@cgmh.org.tw (C.-F.C.); freewind_05@yahoo.com.tw (P.-W.H.); js1101@cgmh.org.tw (J.-S.C.); f22338@cgmh.org.tw (Y.-S.H.); 2 Division of Hematology-Oncology, Department of Internal Medicine, Chang Gung Memorial Hospital at Kaohsiung, Kaohsiung 833, Taiwan; chenyy@cgmh.org.tw; 3 Division of Hematology-Oncology, Department of Internal Medicine, Chang Gung Memorial Hospital at Chiayi, Chiayi 612, Taiwan; luchanghsien@gmail.com; 4 Division of Hematology-Oncology, Department of Internal Medicine, Chang Gung Memorial Hospital at Keelung, Keelung 204, Taiwan; ph555chang@cgmh.org.tw

**Keywords:** gemcitabine, pancreatic cancer, Taiwan, prognostic model, overall survival

## Abstract

Gemcitabine plus S-1 (GS) is commonly used to treat advanced pancreatic cancer (APC) in Asia. Few clinical experiments have demonstrated the clinical efficacy of GS in routine clinical practice. We aimed to identify the prognostic factors and develop a prognostic model for survival prediction in patients with APC, treated with GS. Records of 111 patients with newly diagnosed APC who received first-line palliative GS chemotherapy during 2010–2016 in Taiwan were analyzed retrospectively. Univariate and multivariate analyses were performed for the identification of prognostic factors. A prognostic model using prognosticators from the multivariate analysis was developed for survival prediction. The median overall survival (OS) for the cohort was 9.3 months (95% confidence interval [CI], 8.0–10.6). The prognostic model was constructed based on four independent prognosticators: performance status, tumor stage, pre-treatment albumin level, and neutrophil-to-lymphocyte ratio. Patients were categorized by tertiles into good, intermediate, and poor prognostic groups. The median OS values for each of these groups were 21.1 (95% CI, 8.2–33.9), 9.2 (95% CI, 8.3–10.1), and 5.8 months (95% CI, 4.4–7.1; log-rank *p* < 0.001), respectively. The bootstrapped corrected C-index of this model was 0.80 (95% CI, 0.71–0.89). The developed model was robust and could accurately predict survival in this population, and can assist clinicians and patients in survival discrimination and the determination of appropriate medical care goals. Additional research is needed to externally validate the model’s performance.

## 1. Introduction

Pancreatic cancer is the seventh leading cause of cancer-related death, worldwide [1], and eighth in Taiwan [2], with a five-year survival rate of 8.5% in the United States of America [1] and 6.7% in Taiwan [2]. Owing to this disease’s unspecific symptoms and a lack of effective screening tools, most patients have advanced pancreatic cancer (APC), including locally advanced or metastatic disease, at diagnosis [3].

Systemic chemotherapy is the standard treatment for APC [4,5,6,7,8,9,10,11]. The active agents used in the first-line treatment of APC include 5-fluouracil [7], gemcitabine [8], gemcitabine combination with nab-paclitaxel [10], gemcitabine combination with Erlotinib [9], or the FOLFIRINOX (folinic acid, 5-fluorouracil, irinotecan, oxaliplatin) regimen [7]. However, despite treatment with these agents, the median survival time associated with this disease remains 6–11 months [8,9,10,11].

S-1 is an oral 5-fluorouracil derivative that has been widely used in Japan in the treatment of various types of solid cancers since its approval in 1999 [12,13]. The Gemcitabine and S-1 Trial (GEST) study was a randomized phase III study conducted in Japan and Taiwan for the evaluation of the clinical efficacy of S-1 monotherapy, gemcitabine monotherapy, and gemcitabine plus S-1 (GS) as the first-line treatment for APC [14]. In that study, progression-free survival (PFS) time was significantly better in the GS group than the gemcitabine group (5.7 months versus 4.1 months, *p* < 0.001). The median overall survival (OS) time was two-fold higher in the GS group than the gemcitabine group (11.2 months versus 5.3 months) in a subgroup analysis of Taiwanese patients [15], making GS an attractive choice of treatment in Taiwan, especially after the reinstatement of S-1 in June 2014.

A number of parameters with predictive value have been identified in patients with APC, including performance [16,17], clinical symptoms with pain or body weight loss [17,18], tumor location [19], tumor stage [16,18,19], metastatic organ [20], and laboratory biomarkers [16,17]. However, the information regarding the effect of clinical variables on survival was inconsistent and varied widely due to the heterogeneous treatment modalities with different chemotherapeutic regimens. To date, few clinical experiments have demonstrated the clinical efficacy, or demonstrated the known prognostic factors of GS in routine medical care. This study was performed for the evaluation of the prognostic factors associated with the GS regimen in the first-line treatment of APC in Taiwan; additionally, we aimed to develop a prognostic model for the prediction of survival outcomes in patients with pancreatic cancer treated with GS.

## 2. Results

### 2.1. Patient Characteristic and GS Treatment Duration

Data on the patients’ characteristics and laboratory values are shown in Table 1. The median age of the 111 patients was 62 years (range, 32–82 years), and 59.5% of them were men. A total of 80.2%, 17.1%, and 2.7% patients had an Eastern Cooperative Oncology Group performance scale (ECOG PS) score of 0–1, 2, and 3, respectively. In total, 22 (19.8%) patients had a locally advanced disease (stage III), whereas 89 (80.2%) had a metastatic disease (stage IV). The most common site of metastasis was the liver (51.4%), followed by the peritoneum (27.9%), distal lymph nodes (15.3%), and lungs (11.7%).

The median duration of GS treatment was 4.7 months. The median relative dose-intensity was 71% for gemcitabine and 78% for S-1. In all, 45 (40.5%) patients received second-line chemotherapy after disease progression with the GS regimen.

### 2.2. Survival and Prognostic Score

**Figure 1:**
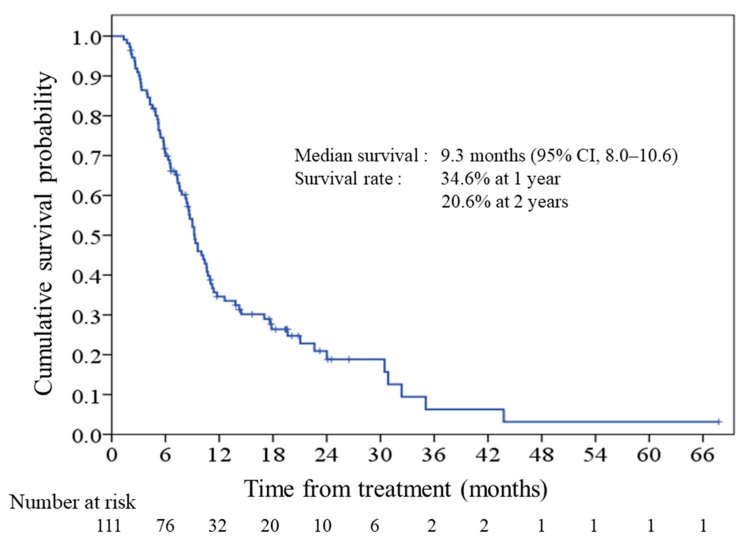
Kaplan-Meier plot of overall survival in patients with advanced pancreatic cancer treated with gemcitabine plus S-1. CI, confidence interval.

The median overall survival for the cohort was 9.3 months (95% CI, 8.0–10.6), as shown in Figure 1, and 27 (24.3%) of the patients were alive at the end of the study. The associations between clinical variables and OS in the univariate and multivariate analyses are shown in Table 2. ECOG PS, tumor stage, pre-treatment albumin level, and the neutrophil-to-lymphocyte ratio (NLR) were the most significant prognostic factors for OS in the multivariate analysis. *β*-coefficients of the prognostic factors defined by multivariate Cox modeling are shown in Table 3. The final prognostic model based on the multivariate analysis is:

Prognostic score = 3 + (0 if ECOG PS 0 or 1; 0.359 if ECOG PS 2; 2.709 if ECOG PS 3) + (0 if stage III; 1.636 if stage IV) + 0.186 × NLR (100%) − 0.547 × albumin (g/dL)

**Figure 2:**
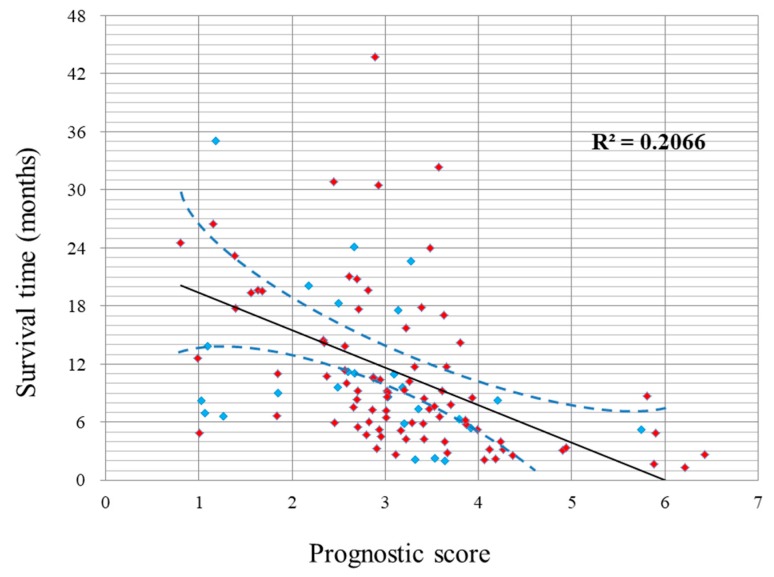
Scatter plot showing a linear correlation between prognostic score and survival time. The red color indicates the patients who died and the blue color indicates those who were alive at the end of the follow-up. The dashed line indicates the 95% confidence interval of the survival time for each score.

Patients with a higher prognostic score had a shorter survival time. The linear correlations between the prognostic scores and survival times are shown in Figure 2 (R square = 0.21).

### 2.3. Performance of the Prognostic Model

**Figure 3:**
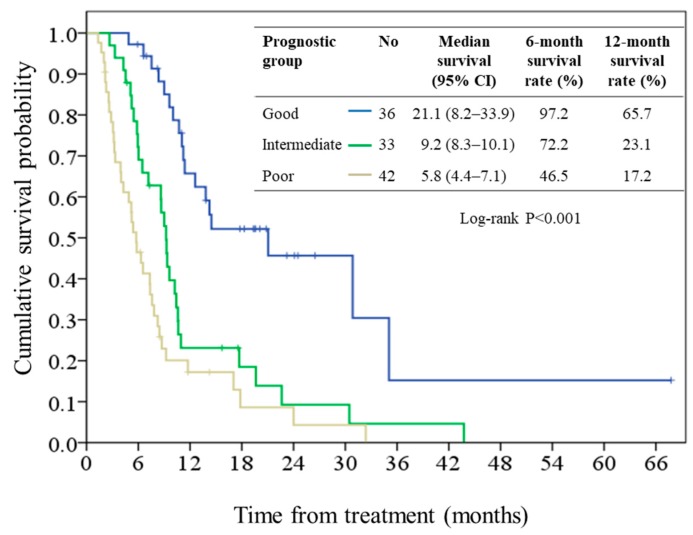
Kaplan-Meier plot of overall survival in patients stratified by prognostic group. CI, confidence interval.

Using the developed model, all patients were categorized by tertiles into the good (prognostic score <2.7), intermediate (prognostic score 2.7 to 3.3), and poor prognostic groups (prognostic score >3.3) as shown in Table 4. The median OS values for each of these groups were 21.1 months (95% CI, 8.2–33.9), 9.2 months (95% CI, 8.3–10.1), and 5.8 months (95% CI, 4.4–7.1; log-rank *p* < 0.001), respectively, as shown in Figure 3. The hazard ratio was 2.80 (95% CI: 1.55–5.06; *p *=** 0.001) when comparing the intermediate and good prognostic groups, and 4.81 (95% CI: 2.71–8.56; *p* < 0.001) when comparing the poor and good prognostic groups. The bootstrapped corrected C-index of this model was 0.80 (95% CI, 0.71–0.89).

## 3. Discussion

Pancreatic cancer is a lethal malignancy with a modest response to systemic chemotherapy [21]. Although GS treatment is associated with a higher response rate [14], and better PFS [14] and OS [22] than gemcitabine in APC [19,20], a substantial proportion of our patients still had progressive disease and, therefore, were exposed to unnecessary toxicity. Using patients’ clinical variables in conjunction with their laboratory test values, we developed a prognostic model for the prediction of survival outcomes in patients with APC who underwent palliative chemotherapy with GS. The model accurately predicted the survival probability with a bootstrapped corrected C-index of 0.80.

An important difference between our patient cohort and that of the GEST study is that our patients showed a higher proportion of metastatic disease (80.2% in our study versus 75.7% in the GEST study) and poorer PS (80% of our patients versus 100% of those in the GEST study had an ECOG PS of 0 or 1), resulting in our study showing poorer outcomes (median OS, 9.3 months) than the GS arm in the GEST study (10.1 months) [14]. In the present study, we explored the prognostic factors related to APC treated with GS based on data obtained from four institutes across Taiwan. Using our prognostic model, all patients who received GS therapy could be simply categorized into three different prognostic groups for the identification of those who may experience the strongest benefit and the selection of those showing the lowest clinical treatment efficacy.

Our prognostic model may help clinicians to discuss the choice of regimens with their patients. Patients in the good prognostic group could achieve the best median overall survival of 21.1 months. In our opinion, clinicians should encourage these patients to receive combination chemotherapy (such as the GS regimen in our study) with the aim to reach the longest overall survival. In contrast, clinicians might consider providing monotherapy, or the best supportive care for patients in the poor prognostic group, as they have the lowest efficacy from the GS combination treatment.

Regarding the calculation of the prognostic model, the sum score was calculated as follows: constant plus *β*-coefficient, multiplied by the value of each variable, depending on its presence or absence. Constants are the same for all patients, whereas the variable values are specific to each patient. An example of the calculation for three hypothetical patients is provided in Table 5. If a given patient with ECOG PS 0, stage III disease, NLR of 3, and albumin level of 4.0 g/dL, then his sum of scores would be: 3 + 0 (ECOG PS 0) + 0 (stage III) + 0.186 × 3 (NLR) − 0.547 × 4.0 (albumin level) = 1.37. Therefore, this patient would be categorized in the good prognostic group.

Some of the clinical variables in our prognostic model are well-known prognosticators in APC, such as PS and tumor stage (locally advanced versus metastatic). Patients with APC treated with systemic chemotherapy had poorer outcomes if their PS was 2–3 compared to those with a PS of 0–1 [23]. A previous report showed that the possibility of one-year survival decreased from 11% in locally advanced disease to 5% in metastatic disease [24]. Similarly, patients with locally advanced stage pancreatic cancer had better survival outcomes than those with metastatic disease in our study. The impact of tumor stage on survival is not only influenced by a smaller tumor burden, but also the better GS efficacy in locally advanced disease than metastatic disease [14].

Kurihara et al. developed a prognostic model using performance status, tumor stage, and absolute neutrophil count, in the prediction of survival outcome based on 182 patients with unresectable pancreatic cancer, treated with gemcitabine or S-1 monotherapy [16]. Similar to Kurihara’s model, our prognostic score adds on another two important clinical variables, including albumin and NLR. Albumin is frequently used as a biomarker of nutritional and inflammatory processes in cancer [25,26,27,28]. An elevated NLR is linked to increased neutrophil mobilization and inflammatory response to tumor-secreted cytokines, and correlated with poor prognoses in cancer [29,30,31,32,33]. However, there is no consensus on the optimal albumin and NLR cut-off values for prognoses. NLR values ranging from >2 to >5 were arbitrary allocated as cut-off values for prognoses in previous reports [34,35,36,37]. The variations in the cut-off values across different studies may impede the utility of albumin or the NLR as prognosticators in clinical practice. To overcome this shortcoming, our model used the continuous variables of albumin and the NLR as prognostic factors. We believe our model is easier to use in clinical practice and may provide further survival discrimination in patients with different albumin or NLR values.

To the best of our knowledge, this is the first prognostic model developed for the prediction of survival outcomes in APC patients who underwent palliative chemotherapy with GS. This study’s strengths lie in its inclusion of a relatively large number of patients. Furthermore, all the clinical variables in our model are easy to access; therefore, this model has scope for wide applicability at the time of antitumor therapy initiation. However, our study has some limitations. First, selection bias existed owing to the retrospective design of the study. Some of the patients received GS before the reimbursement of S-1 in June 2014, and this may have led to the selection of those with better financial resources or family support. Second, as our model was developed based on APC patients treated with GS as first-line systemic chemotherapy, its applicability may be limited in other countries, in which GS is not the treatment of choice in first-line settings for APC. Finally, and most importantly, this model was internally validated using the bootstrapping method; external validation is mandatory before this model can be used, worldwide. In addition, it is worth testing the utility of this prognostic model on patients treated by gemcitabine, gemcitabine + Nab-paclitaxel, or the FOLFIRINOX regimen, which are standard chemotherapy regimens in other (non-Asian) countries.

## 4. Materials and Methods

### 4.1. Patients and Treatment

This study included 111 patients with newly diagnosed APC between 2010 and 2016 at the four institutes of the Chang Gung Memorial Hospital (CGMH) in Taiwan. All patients had either a pathological or radiographical diagnosis of primary pancreatic cancer, and had received a GS regimen as the first-line treatment for pancreatic cancer. Patients who had other active concurrent malignancies, neuroendocrine tumors, or histological type sarcomas, and received concomitant radiotherapy during GS therapy were excluded. Gemcitabine (1000 mg/m^2^) was administered on days one and eight, whereas S-1 (60–100 mg) was administered daily on days one to 14 every 21 days, per the GEST study [19]. The exact treatment dosage was decided by the primary care physician. Patient characteristics were analyzed for the identification of the variables associated with survival outcomes. This study was approved by the institutional review boards of all the CGMH branches on 22 November 2017 (ethic code: 201701796B0) and has been conducted in compliance with the Helsinki Declaration (1996).

### 4.2. Data Collection and Follow-Up

Administrative and clinical data comprised of patient demographics collected retrospectively, including clinical features, with age, sex, and Eastern Cooperative Oncology Group performance status (ECOG PS); pre-existing comorbidities; pretreatment body mass index (BMI); pretreatment complete blood count, biochemistry, carcinoembryonic antigen (CEA) level; and cancer antigen 19-9 (CA19-9) level within seven days of GS therapy initiation. The neutrophil-to-lymphocyte ratio (NLR) was calculated by dividing the neutrophil count by the lymphocyte count. Comorbidities were represented by the modified Charlson comorbidity index (CCI) [38,39], excluding patient age and cancer diagnosis. OS was calculated from the time of GS combination therapy initiation until the date of death from any cause. All included patients were followed-up until death or 31 December 2017. All dates of death were obtained from either the Institutional Cancer Registry or the National Registry of Death database in Taiwan.

### 4.3. Statistical Analysis

Basic demographic data were summarized as *n* (%) for categorical variables and median with 95% confidence intervals (CIs) for continuous variables. Clinical variables potentially associated with OS were examined through univariate logistic regression. Variables with *p* < 0.10 in the univariate analysis were included in the multivariate model. A multivariate proportional hazard Cox model with backward selection was used to determine the factors that were independently predictive of survival. The prognostic score was selected according to the sum of constant and *β*-coefficient from each independent factor. The predicted OS probability for each patient was obtained using the prognostic model. Subsequently, all patients were arbitrary categorized by tertiles into the good, intermittent, and poor prognostic groups according to their total scores for survival comparison. Survival time was analyzed using the Kaplan–Meier method. Log-rank tests were used to determine significant differences between the survival curves. The prognostic model was internally validated using the bootstrapping method (1000 repetitions) to obtain the C-index for the assessment of model performance. All statistical assessments with *p* < 0.05 were considered significant.

## 5. Conclusions

The present study showed that the use of GS as the first-line therapy in routine clinical practice yielded similar survival outcomes in APC patients as those observed in the GEST study. Our study identified ECOG PS, tumor stage, pre-treatment albumin level, and the NLR as being independent prognosticators for APC patients receiving GS. Accordingly, we developed a robust prognostic model that accurately predicted survival in patients with APC who underwent palliative chemotherapy with GS. This model may assist clinicians and patients in survival discrimination and the determination of appropriate medical care goals. Additional research is needed to externally validate the performance of this model.

## Figures and Tables

**Table 1:** Patient characteristics (*n* = 111).

Characteristic	Value (%)
Median age, year (range)	62 (32–82)
Male sex	66 (59.5)
Median BMI, kg/m^2^ (range)	22.5 (15.6–32.5)
ECOG PS	
0 or 1	89 (80.2)
2	19 (17.1)
3	3 (2.7)
Charlson comorbidity index	
0	31 (27.9)
1	39 (35.1)
2	21 (18.9)
3	17 (15.3)
4	2 (1.8)
5	1 (0.9)
Smoking	
never	56 (50.5)
ever or active	55 (49.5)
Primary tumor site	
head	32(28.8)
body	24 (21.6)
tail	32 (28.8)
overlapping	23 (20.7)
Presence of jaundice	
No	84 (75.7)
Yes	27 (24.3)
Primary tumor size, cm (range)	4.9 (1.8–13.1)
T-classification	
1	2 (1.8)
2	14 (12.6)
3	30 (27.0)
4	65 (58.6)
N-classification	
0	19 (17.1)
1	92 (82.9)
M-classification	
0	22 (19.8)
1	89 (80.2)
AJCC tumor stage	
III	22 (19.8)
IV	89 (80.2)
Site of metastases	
liver	57 (51.4)
peritoneum	31 (27.9)
distant lymph nodes	17 (15.3)
lung	13 (11.7)
others	4 (3.6)

BMI, body mass index; ECOG PS, Eastern Cooperative Oncology Group performance scale; AJCC, American Joint Committee on Cancer; CEA, carcinoembryonic antigen; CA19-9, carbohydrate antigen 19-9; NLR, neutrophil-to-lymphocyte ratio.

**Table 2:** Univariate and multivariate analyses for overall survival.

Variable	Category	Univariate Analysis		Multivariate Analysis	
		HR (95% CI)	*p* Value	HR (95% CI)	*p* Value
Age	per year	1.02 (0.99–1.04)	0.14		
BMI	per kg/m^2^	0.97 (0.91–1.04)	0.41		
Sex	male	1		1	
	female	0.68 (0.44–1.06)	0.090	0.71 (0.42–1.20)	0.200
ECOG PS	0 or 1	1		1	
	2	2.95 (1.70–5.12)	<0.001	1.43 (0.74–2.77)	0.29
	3	4.58 (1.41–14.9)	0.011	15.0 (3.11–72.6)	0.001
CCI	per index	1.15 (0.94–1.40)	0.17		
Smoking	never	1			
	ever	1.39 (0.90–2.15)	0.16		
Tumor site	head	1		1	
	body	0.75 (0.39–1.44)	0.40	1.58 (0.73–3.42)	0.250
	tail	1.77 (1.01–3.08)	0.045	1.85 (0.96–3.59)	0.090
	overlapping	1.14 (0.60–2.15)	0.69	1.95 (0.93–4.06)	0.080
Presence of jaundice	no	1			
	yes	1.35 (0.80–2.25)	0.26		
Primary tumor size	per cm	1.08 (0.94–1.25)	0.15		
T-classification	1	1			
	2	1.74 (0.23–13.4)	0.600		
	3	1.14 (0.15–8.45)	0.90		
	4	0.60 (0.081–4.44)	0.62		
N-classification	0	1			
	1	1.57 (0.90–2.73)	0.19		
M-classification	0	1		1	
	1	4.57 (2.19–9.54)	<0.001	5.13 (2.05–12.8)	0.001
Hemoglobin, g/dL	>12	1			
	≤12	1.39 (0.58–2.17)	0.14		
Platelet count, 10^9^/L	≥150	1			
	<150	0.99 (0.57–1.71)	0.99		
Leukocyte count, 10^9^/L	<11,000	1			
	≥11,000	1.36 (0.93–2.11)	0.18		
AST, U/L	≤34	1			
	>34	1.02 (0.65–1.60)	0.93		
Alkaline phosphatase, IU/L	≤140	1			
	>140	1.43 (0.91–2.24)	0.13		
CEA, ng/mL	≤5	1			
	>5	1.16 (0.75–1.79)	0.51		
CA19-9, u/mL	≤37	1			
	>37	0.87 (0.52–1.46)	0.60		
Albumin, g/dL	per gm/dL	0.52 (0.33–0.84)	0.008	0.58 (0.33–0.98)	0.043
NLR	per ratio	1.15 (1.07–1.23)	<0.001	1.20 (1.11–1.31)	<0.001

BMI, body mass index; ECOG PS, Eastern Cooperative Oncology Group performance scale; CCI, Charlson comorbidity index; AST, aspartate transaminase; CEA, carcinoembryonic antigen; CA19-9, carbohydrate antigen 19-9; NLR, neutrophil-to-lymphocyte ratio; HR, hazard ratio; CI, confidence interval.

**Table 3:** *β*-coefficient of the prognostic factors defined by multivariate Cox modeling.

Factor	*β*	SE	*p*	Hazard Ratio	95% CI
ECOG PS					
0 or 1	Reference				
2	0.359	0.337	0.286	1.432	0.74–2.77
3	2.709	0.804	0.001	15.0	3.11–72.6
Tumor stage					
III	reference				
IV	1.636	0.469	0.001	5.13	2.05–12.9
NLR, 100%	0.186	0.044	<0.001	1.204	1.11–1.31
Albumin, g/dL	−0.547	0.282	0.043	0.579	0.33–0.98
Constant	3	0.911	0.001	0.041	-

*β*, *β*-coefficient; SE, standard error of the *β*-coefficient; *p*, probability value; 95% CI, 95% confidence interval of the hazard ratio.

**Table 4:** Survival time of patients by prognostic group.

Prognostic Group	Prognostic Score	*N* (%)	Median Survival, Months (95% CI)	Hazard Ratio (95% CI)	*p* Value
Good	< 2.7	36 (32.4)	21.1 (8.2–33.9)	1 (reference)	
Intermediate	2.7 to 3.3	33 (29.7)	9.2 (8.3–10.1)	2.80 (1.55–5.06)	0.001
Poor	>3.3	42 (37.8)	5.8 (4.4–7.1)	4.81 (2.71–8.56)	<0.001

CI, confidence interval.

**Table 5:** Example of prognostic score calculation for 3 hypothetical patients.

Patient	ECOG PS	Stage	Neutrophil	Lymphocyte	NLR	Albumin (g/dL)	Sum of Scores	Prognostic Group
A	0	III	60	20	3	4.0	1.37	good
B	0	IV	60	15	4	4.0	2.916	intermediate
C	3	IV	60	12	5	3.0	3.634	poor
